# Synthesis and Characterization of Photocurable Difunctional Monomers for Medical Applications

**DOI:** 10.3390/polym16243584

**Published:** 2024-12-21

**Authors:** Gokhan Demirci, Agata Goszczyńska, Martyna Sokołowska, Marek Żwir, Krzysztof Gorący, Miroslawa El Fray

**Affiliations:** 1 Department of Polymer and Biomaterials Science, Faculty of Chemical Technology and Engineering, West Pomeranian University of Technology in Szczecin, Al. Piastów 45, 70-311 Szczecin, Poland; gokhan.demirci@zut.edu.pl (G.D.); agata.goszczynska@zut.edu.pl (A.G.); martyna.sokolowska@zut.edu.pl (M.S.); marek.zwir@zut.edu.pl (M.Ż.); krzysztof.goracy@zut.edu.pl (K.G.); 2 Poltiss Spółka z o.o., Al. Wojska Polskiego 150/1, 71-324 Szczecin, Poland

**Keywords:** dibutyltin dilaurate, fatty acid derivatives, telechelic macromonomers, photopolymerization, 3D printing

## Abstract

Photocurable materials offer a rapid transition from a liquid to a solid state, and have recently received great interest in the medical field. However, while dental resins are very popular, only a few materials have been developed for soft tissue repair. This study aims to synthesize a difunctional methacrylate monomer using a dibutyltin dilaurate which is suitable for the photocuring of soft materials. These soft materials were compared with PhotoBioCure^®^ (Szczecin, Poland) material with a similar molecular weight, of Mn ~7000 g/mol on average. Infrared spectroscopy was used to monitor the two-step synthesis catalyzed with dibutyltin dilaurate, while spectroscopic and chromatographic methods were used to determine the chemical structure and molecular weight of the monomers. Photopolymerization kinetics under varying light intensities were explored in a nitrogen atmosphere for representative difunctional monomers. The mechanical testing of the resulting elastomeric films confirmed tensile strength and modulus values consistent with soft tissue parameters in the range of 3–4 MPa. The 3D printability of the macromonomers was also assessed. Additionally, cytotoxicity assessments using cultured cells showed a high cell viability (97%) for all new materials. Overall, we demonstrate that difunctional methacrylate monomers converted to flexible solids during photopolymerization show great potential for biomedical applications.

## 1. Introduction

In recent years, the advancement of injectable materials technology has been a focal point in biomaterials development, particularly for use in soft tissue engineering applications [1,2,3]. This technology allows for the in situ formation of hydrogels and elastomers, which are critical for applications in wound healing, tissue engineering and other therapeutic interventions [4,5,6]. The elastomers that lie behind this technology, known for their flexible and durable properties, are essential materials in the biomedical field, comprising a market that is projected to reach over USD 20 billion by 2026 [1]. They have become vital in procedures such as cardiac repair and hernia repair, where customizable, biocompatible, and minimally invasive solutions are in high demand [7,8,9,10,11,12,13].

Central to the success of these injectable materials is the ability to precisely control their synthesis and properties to meet diverse clinical needs [6,14,15]. Among the various approaches to achieve this, photopolymerization has emerged as a transformative technique [16,17,18,19]. This process, which uses light to initiate and propagate polymerization reactions, offers unparalleled control over curing kinetics and spatial precision, enabling the rapid formation of robust and tailored material networks under physiological conditions [20,21]. Such capabilities make photopolymerization particularly well suited for the development of injectable biomaterials, where on-demand curing is essential for minimally invasive procedures [22,23,24].

A significant challenge in optimizing photopolymerizable materials is the selection of appropriate catalysts and photoinitiators that balance reaction efficiency with biocompatibility. Traditionally, organotin catalysts, such as dibutyltin dilaurate (DBTDL), have been used extensively in polymerization reactions [25,26,27,28,29]. Our previous studies have demonstrated the potential of alternative metal-based catalysts, such as bismuth salts or zinc (II) acetylacetonate, which improved the kinetics of photopolymerization. These catalysts have shown promise in achieving rapid curing under UV light while maintaining the biocompatibility of the resulting hydrogels and elastomers [30].

The versatility of elastomers derived from fatty acid-based macromonomers makes them particularly attractive for soft tissue applications, such as hernia repair and other minimally invasive techniques [31,32]. Achieving the appropriate balance between mechanical strength and elasticity is crucial for such applications, as the materials need to withstand physiological stresses while integrating with soft tissues [13,33,34,35,36,37]. The customization of these materials, whether by tuning their molecular structure or optimizing the curing conditions, is an important step toward addressing the diverse needs of biomedical procedures. Through the careful selection of catalysts and polymerization conditions, it is possible to tailor the degradation rate, mechanical properties, and biocompatibility of the materials.

**Scheme 1:**
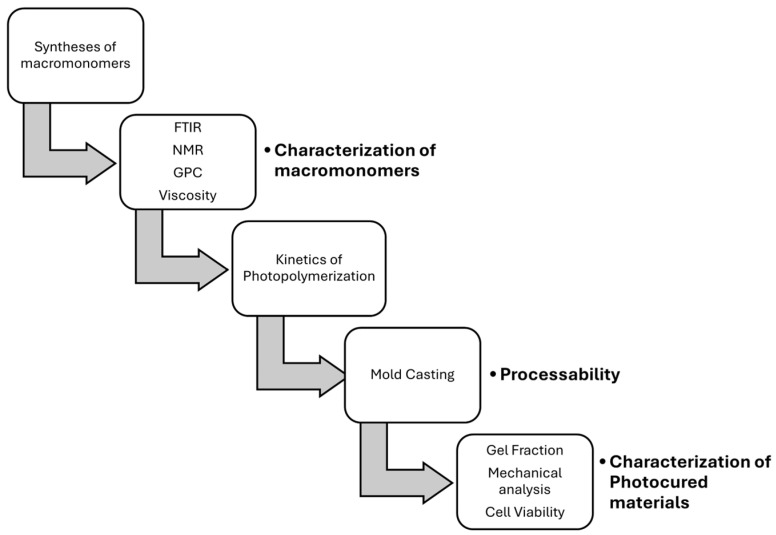
Schematic diagram of the study.

In this work, we focus on optimizing the manufacturing process of photocurable elastomer networks by investigating the use of dibutyltin dilaurate catalysts, particularly through the fine-tuning of curing conditions. Our approach emphasizes the customization of material properties to match the specific requirements of soft tissue repair, such as hernia repair, where flexibility, strength, and biocompatibility are essential. Additionally, we have explored the 3D printing capabilities of these elastomers, assessing their printability to create precise, custom structures (Scheme 1). The novelty of this work lies in the integration of process optimization with application-specific material customization and advanced fabrication techniques, bridging the gap between material science and clinical needs. The results of this study are expected to contribute to the further advancement of photocurable materials in the biomedical industry, offering improved solutions for minimally invasive procedures.

## 2. Materials and Methods

### 2.1. Materials

Priplast 1838, which is a polyester polyol, was provided from Cargill Bioindustrial (Gouda, The Netherlands). Dibutyltin dilaurate (DBTDL) and phenothiazine (PTZ) were purchased from Sigma-Aldrich (Poznań, Poland). 2-hydroxyethylmethacrylate (97%) (HEMA) and isophorone diisocyanate (98%) (IPDI) were purchased from Merck KGaA (Darmstadt, Germany). A Photoinitiator Omnirad 2022 was purchased from IGM Resins (Waalwijk, The Netherlands). Dichloromethane (DCM), ethanol and ethyl acetate (EtOAc) were purchased from Chempur (Piekary Śląskie, Poland). Hydrochloric acid (HCl) and the murine fibroblast cell line L929, along with all cell culture reagents, were obtained from Sigma-Aldrich (Poznań, Poland). HEMA was utilized following distillation under reduced pressure, while all other reagents were employed without further purification. PhotoBioCure^®^ difunctional monomer of Mn = 7000 g/mol was provided by Poltiss sp. z o.o. (Szczecin, Poland) and used as a reference material.

### 2.2. Synthesis of Difunctional Macromonomers with DBTDL

**Figure 1:**
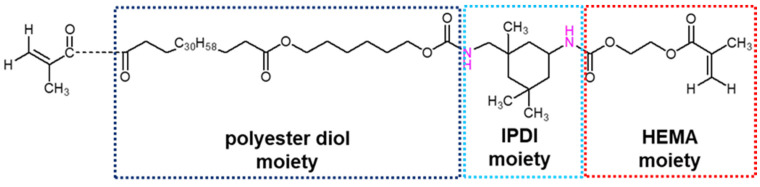
Structure of the synthesized difunctional methacrylate monomer containing ester and urethane groups.

The process consists of two following steps to obtain the material with the structure shown in Figure 1.

Before the synthesis, the flask was washed in an alkaline bath, rinsed with distilled water, 0.1 M HCl solution, acetone and DCM, then dried at room temperature. 50 mL of DCM was introduced in a 250 mL round-bottom flask that was kept in an ice bath after three Argon/Pump cycles. DABCO (23.04 mg, 0.21 mmol) in 7 mL of DCM was added into the flask. Then, IPDI (11.0 mL, 51.70 mmol) was dosed with the help of a syringe pump (speed 66 mL/h), using a dicoNEX syringe (20 mL). At the same time, Priplast 1838 (50.03 g, 51.73 mmol) was dissolved in 50 mL of DCM, placed in a dropper and instilled dropwise into the ice-cold reaction mixture (dosing time of about 45 min). After completing the addition, the flask was placed in an oil bath, and the reaction was maintained at 35 °C. The reaction progress was monitored using FT-IR spectroscopy by tracking the ratio of absorbance at 2262 cm^−1^ (A_2262_), corresponding to the N=C=O vibrations of isocyanate groups in IPDI, to absorbance at 1526 cm^−1^ (A_1526_), associated with the N-H bending vibrations of the formed urethane bonds. The first step was deemed complete once the NCO/NH ratio stabilized, which was typically between 4 and 5, indicating that 50% of the -NCO groups had been consumed. In the second step, PTZ (4.09 mg, 0.02 mmol) in 7 mL of DCM and DBTDL (63.49 mg, 0.10 mmol) in 7 mL DCM were introduced, respectively. Subsequently, HEMA (13.0 mL, 106.88 mmol) was added using a syringe pump at a controlled rate of 78 mL/h, with the reaction shielded from light. Completion of the reaction was confirmed by FT-IR spectroscopy, indicated by the disappearance of the 2262 cm^−1^ band and the A2262/A1526 ratio reaching zero, signifying the full consumption of -NCO groups. The flask was then removed from the oil bath and allowed to cool to room temperature. The product was precipitated six times into a sixfold excess of ice-cold methanol, with re-dissolution in DCM (50 mL) for each cycle. Residual solvent was removed under reduced pressure at 50 °C. To verify the absence of residual solvents (MeOH and DCM), ^1^H-NMR spectroscopy was conducted. The final product was stored in an amber-colored flask at 4 °C.

### 2.3. Photocuring Process

Preparation of crosslinking mixture as follows: materials (DBTDL-catalyzed monomer and PhotoBioCure^®^ material) were dissolved in ethyl acetate (EtOAc) (mg/mL) and 0.5, 1, 1.5, 2 and 2.5 wt.% photoinitiator (Omnirad 2022, IGM Resins B.V., Waalwijk, The Netherlands) in 5 mL EtOAc was added into flask while protecting the product from the light. To confirm the absence of residual EtOAc, the solvent was evaporated under reduced pressure after achieving a homogeneous mixture. Subsequently, 1-mm-thick films were created by pouring the final composition onto a glass plate and spreading it evenly with a steel applicator. The composition was then exposed to irradiation using a DYMAX Bluewave LED Prime UVA light source (Dymax Corporation, Torrington, CT, USA), featuring a narrow spectral range with a maximum intensity at a wavelength of 385 nm. The radiation intensity was set to 20 mW/cm^2^ using an AktiPrint radiometer (Technigraf GmbH, Grävenwiesbach, Germany). Photocrosslinking was conducted under argon within a glove box. Each spot (2.25 cm^2^) was exposed for 150 s, with the process performed stepwise across the entire plate (10 cm × 20 cm).

### 2.4. Mold Casting

**Figure 2:**
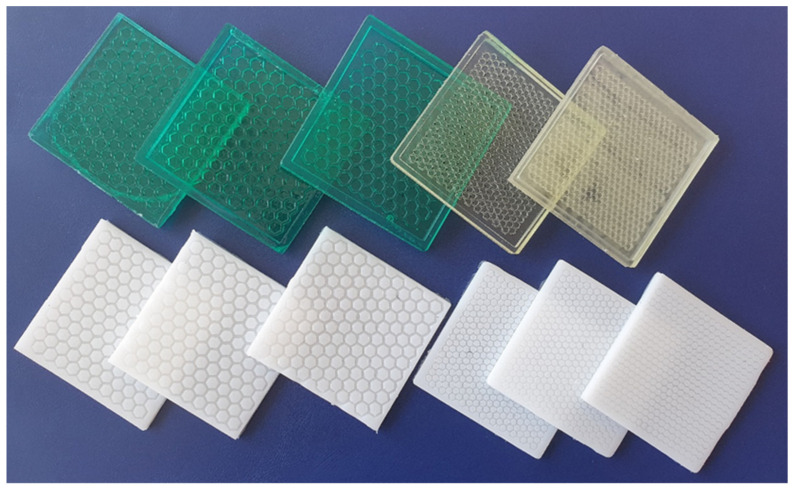
Various versions of the positive matrices are shown—3D printed molds using the SLA technique in the top row, and the negative molds made by RTV silicone rubber in the bottom row.

The practical possibilities of producing meshes using the open mold casting technique, followed by UV light curing of the castings, were investigated according to the following process: (i) development of a positive matrix design representing both the final shape of the mesh and additional elements needed to produce negative molds from this matrix; (ii) creation of a negative mold (or multiple molds), i.e., a production mold, using the positive matrix (Figure 2). In a flexible silicone mold, which was made using a positive matrix prepared with SLA-LCD 3D printing technology from designated photocurable rigid resin, tests were conducted at 23 °C using difunctional monomers to produce thin meshes with a mesh size up to 4 mm in a hexagonal configuration, followed by cross-linking under UV light (λ_max_ of 385 nm and radiation intensity: 20 mW/cm^2^).

### 2.5. Characterization Methods

Fourier transform infrared spectroscopy (FT-IR) of DBTDL-catalyzed materials was performed using a BRUKER ALPHA Platinum spectrometer (Karlsruhe, Germany) at room temperature. Measurements covered the 4000–600 cm^−1^ range with a resolution of 2 cm^−1^ and 32 scans. Liquid monomers were analyzed in transmission mode by placing samples between NaCl plates, while spectra of photocrosslinked films were obtained in reflection mode using an ATR snap-in accessory with a diamond crystal. Data analysis was conducted with EZ OMNIC v7.3 software.

The nuclear magnetic resonance (NMR) spectra of difunctional monomers was recorded on a Bruker DPX HD-400 MHz instrument (Billerica, MA, USA) equipped with a 5 mm Z-gradient broadband decoupling inverse probe at 25 °C. Approximately 20 mg of each monomer was dissolved in 0.7 mL of CDCl_3_-*d*, with naphthalene serving as an internal standard.

Gel permeation chromatography (GPC) was utilized to determine the molecular weights and distributions of the difunctional monomers. The setup included a Wyatt (Dernbach, Germany) instrument, a guard column, four PSS columns (50, 100, 1000, and 100,000 Å), a WGE Dr. Bures Dn 2010 differential refractometer (RI)(Wyatt, Dernbach, Germany), and a Wyatt MALLS DAWN HELEOS multi-angle light scattering (LS) detector (Wyatt, Dernbach, Germany). Measurements were conducted in THF at 35 °C with a flow rate of 1 mL/min. Molecular weight parameters were calculated using calibration, with eight linear polystyrene standards (molecular weights ranging from 970 to 126.700 g/mol). Prior to injection, sample solutions were filtered through a 0.20 µm SRP 20 filter (Bionovo, Legnica, Poland). Data processing was performed with PSS WinGPC Unity (Version: 8.33) software.

To evaluate the gel fraction, crosslinked samples were first weighed (W_initial_), refluxed for 6 h in a Soxhlet apparatus (Behr Labor-Technik, Düsseldorf, Germany) with EtOAc as the solvent, and then dried under reduced pressure to a constant weight (W_final_). The gel fraction was calculated using Equation (1).

Gel fraction (%) = W_final_/W_initial_ × 100 (1)

Dynamic viscosity tests of difunctional monomers were performed using a BROOKFIELD AMETEK rotary rheometer ((Middleboro, MA, USA) with a plate–plate system (ϕ = 40 mm, h = 1 mm), at 30% deformation and a constant shear rate of 0.200 s^−1^, at 25 °C.

The UV-Differential Scanning Calorimetry (UV-DSC) study was conducted using a Q2500 DSC (TA Instruments, New Castle, DE, USA) with an OmniCure 2000 Spot UV Curing System (200 watt mercury vapor short arc). Samples weighing 5 mg were mixed with 0.5–2.5 wt.% of photoinitiator (Omnirad 2022) and irradiated at 365 nm under nitrogen atmosphere (20 mL/min flow) at 25 °C, using three intensities: 20, 100, and 500 mW/cm^2^.

The mechanical tensile properties of photocured resins were evaluated with an Instron 3366 (Instron, High Wycombe, UK) testing system (500 N load cell) at a crosshead speed of 25 mm/min. A total of 6 to 10 rectangular samples (10 mm width, 60 mm length, 0.5 mm thickness) were tested for modulus of elasticity (E), calculated at 2–3% and 5–6% elongation, elongation at break (ε_br_), and tensile strength (σ_br_).

The cytotoxicity of photocured resins was assessed using extract tests in accordance with ISO 10993-5 [38]. Strips of photocured materials (3 cm^2^ in area and 0.5 mm thick) were cut into smaller pieces, placed in 24-well plates, and incubated with 1 mL of complete growth medium (DMEM supplemented with 10% FBS, 2 mM L-glutamine, 100 U/mL penicillin, and 100 µg/mL streptomycin) for 24 h at 37 °C in a CO_2_ incubator. The controls included: (1) positive control: nitrile glove (Mercator Nitrylex Classic), (2) negative control: non-toxic poly(ε-caprolactone) (PCL, Capa ™ 6430)(Perstorp Polyols Inc., Toledo, OH, USA), and (3) sham control: empty media. Simultaneously, 10,000 L929 cells (passages 10–25) were seeded in each well of a 96-well plate with complete growth medium. After 24 h, the medium was removed and replaced with 100 µL of the extract medium from the tested materials (5–6 technical replicates, 2 samples per material). Following an additional 24-h incubation, cell viability was evaluated using light microscopy (Delta Optical IB-100, Minsk Mazowiecki, Poland) and the resazurin viability assay. For this, 20 µL of resazurin stock (0.15 mg/mL in PBS) was added to each well, incubated for 4 h at 37 °C, and Biotek Synergy HTX (Winooski, VT, USA) was used as a plate reader. The cell viability was expressed as a percentage of the sham-extract treated cells.

## 3. Results and Discussion

### 3.1. Reaction Progress Based on Infrared Spectroscopy

DBTDL is a common catalyst that is widely used for biomaterials synthesis. Here, we assessed its performance in the synthesis of difunctional methacrylate monomers suitable for photopolymerization with the ultimate goal of producing material suitable for medical applications.

**Figure 3:**
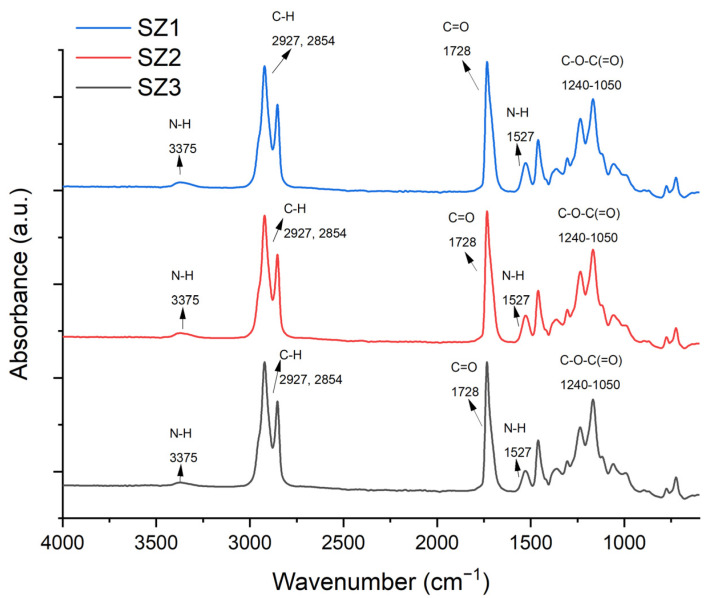
FT-IR spectra of macromonomers (SK_Sn).

Overall, the progress of the reaction for all syntheses performed in triplicate was similar as presented in Table 1 and Figure 3.

As indicated in Table 1, the reaction yields for all the reactions performed were similar, varying between 65% and 68% thus indicating very good reproducibility. The total reaction time lasted from 95 to 98 h, thus showing similarity to data reported for similar systems [39,40].

### 3.2. Chemical Structure of Macromonomers by IR Spectroscopy

FT-IR measurements confirmed that the chemical structures of the synthesized macromonomers were consistent with the expected reaction mechanism. All spectra obtained were similar, indicating that macromonomers had the same chemical structure. The representative FT-IR spectra of the SK1_Sn material are shown in Figure 3. The absorbance bands at 3375 cm^−1^ and 1527 cm^−1^ correspond to N-H stretching vibrations in urethane groups [41]. The band at 1643 cm^−1^ represents C=C stretching vibrations from HEMA coupling [42]. Bands at 2927 cm^−1^ and 2854 cm^−1^ correspond to C-H stretching in -CH_2_ and -CH_3_ groups [43]. The C=O stretching band at around 1728 cm^−1^ in ester bonds of the polyester polyol [44]. The 1460 cm^−1^ band corresponds to -C-C- and R-CH_2_-CH_3_ in rings, while the 1369 cm^−1^ band represents C-H rocking vibrations in fatty acid chains [45]. Bands at 1302 cm^−1^ and 1050 cm^−1^ correspond to RCOOR’ in esters [46], while bands at 1240 cm^−1^ and 1168 cm^−1^ represent -C-O- and -C-O-C(=O) stretching vibrations, respectively [47].

### 3.3. Chemical Structure of Macromonomers by NMR Spectroscopy

**Figure 4:**
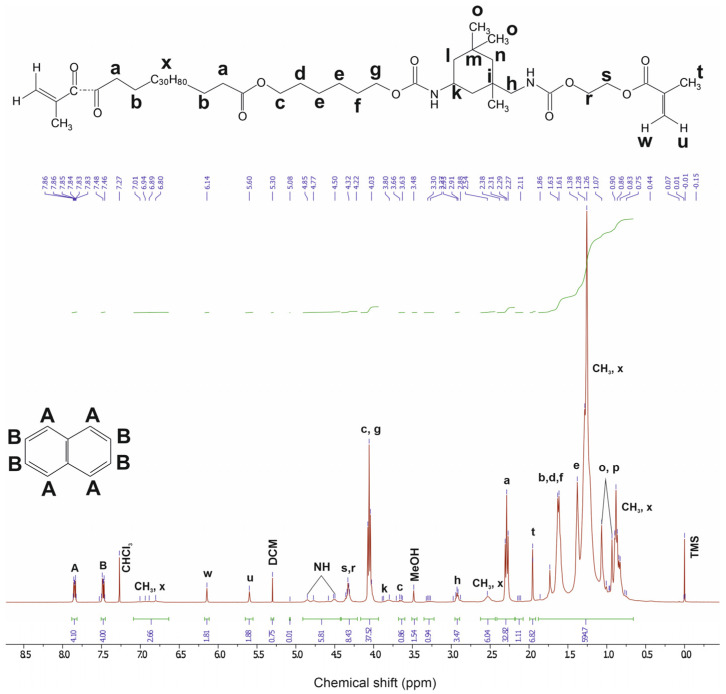
^1^H-NMR spectrum of SK1_Sn.

The chemical structure of the synthesized macromonomers was confirmed through ^1^H NMR spectroscopy. The NMR analysis further revealed that all the obtained macromonomers exhibited similar structures. As a representative example, Figure 4 presents the ^1^H NMR spectrum of the macromonomer synthesized using DBTDL.

The spectra are nearly identical, indicating that the obtained macromonomers are similar and the syntheses are reproducible. In the ¹H NMR spectrum, the peaks at 4.50 and 4.85 ppm correspond to the N-H groups from the urethane bonds [48], while the peaks at 5.60 and 6.14 ppm indicate the presence of C=C groups from the attachment of HEMA [49].

### 3.4. Molecular Mass and Viscosity of Macromonomers

The GPC analysis results are summarized in Table 2. All macromonomers showed a similar Mn ~7000 g/mol (comparable to PhotoBioCure^®^ material), and a dispersity index (D) of 1.64–1.74. Notably, the molecular weights (Mw) of the obtained macromonomers were comparable to those from our previous work [30], despite the use of a different catalyst.

### 3.5. Rheology

Table 3 shows the dynamic viscosity results. It should be noted that materials synthesized with DBTDL are more viscous than PhotoBioCure^®^. The rheological properties of injectable materials are of crucial importance for minimally invasive procedures [50]. They must be sticky enough to stay in place, but not so sticky that they become undeliverable. The high viscosity of the synthesized macromonomers and PhotoBioCure^®^ material suggests they will be well retained at the target site during photocuring. We already demonstrated that, at viscosities below 1000 Pa·s, the macromonomers remain injectable through an 18 G needle [51]. Although detailed clinical delivery systems are beyond the scope of this study, these materials appear well suited for minimally invasive surgeries requiring larger instruments, such as those used in laparoscopic or catheter-based procedures, including hernia or cardiac repair [52].

### 3.6. Photo-DSC Study

**Figure 5:**
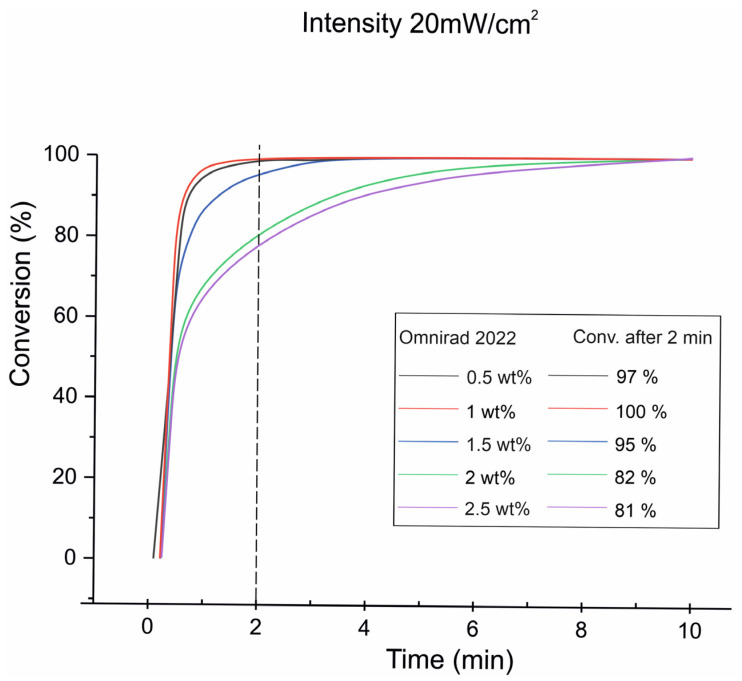
The degree of conversion determined for SK1_Sn compositions cross-linked at an intensity of 20 mW/cm^2^.

**Figure 6:**
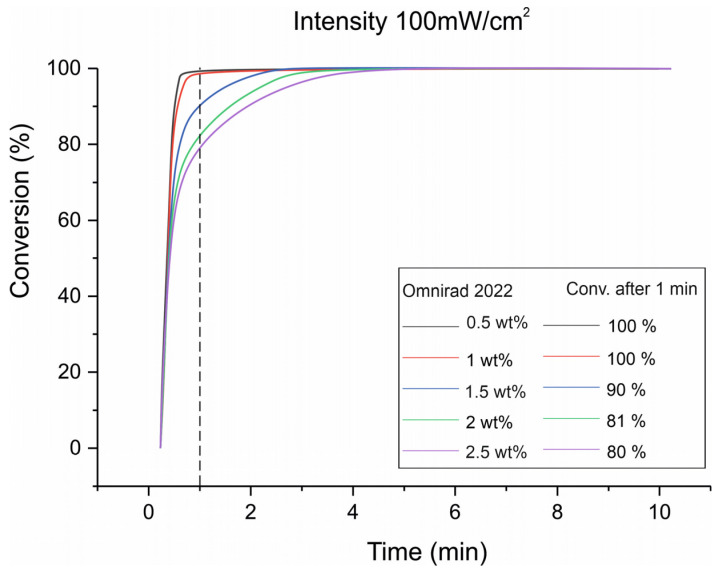
The degree of conversion determined for SK1_Sn compositions cross-linked at an intensity of 100 mW/cm^2^.

**Figure 7:**
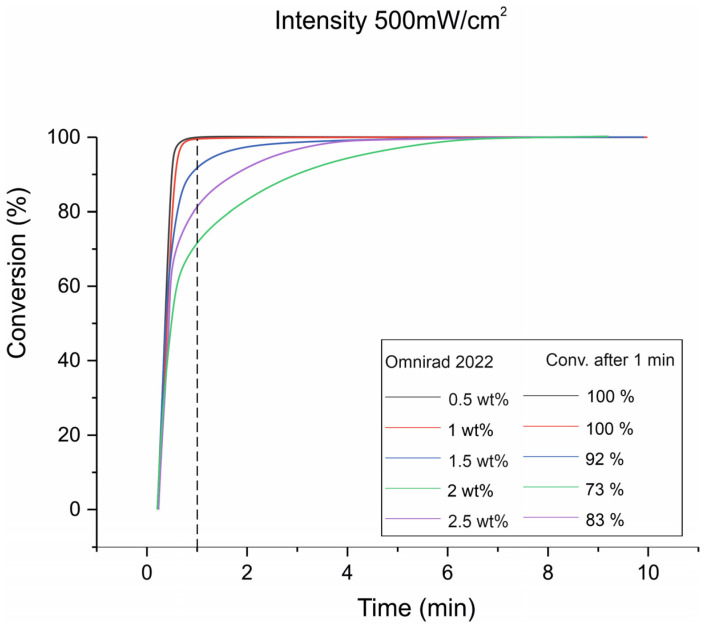
The degree of conversion determined for SK1_Sn compositions cross-linked at an intensity of 500 mW/cm^2^.

**Figure 8:**
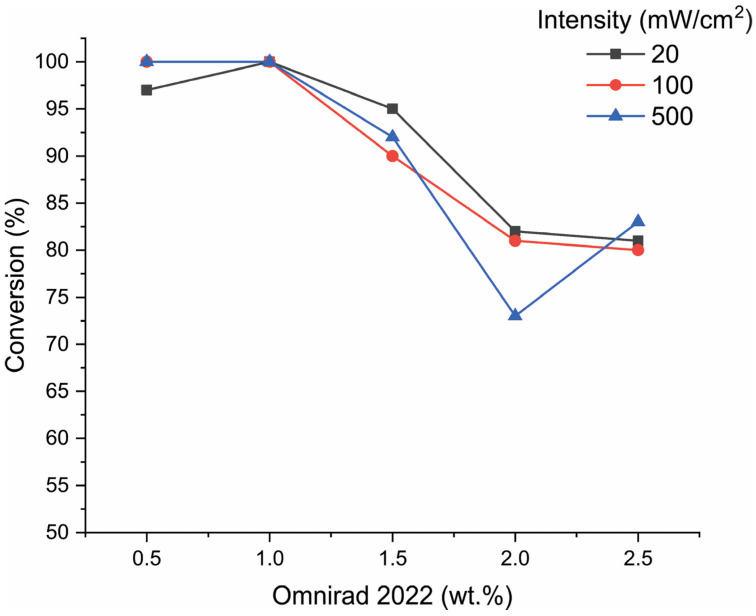
The degree of conversion at 1 min determined for SK1_Sn compositions cross-linked at various light intensities.

For the determination of the optimal conditions and parameters for photopolymerization, as well as the type and concentration of the catalyst, photo-DSC measurements were conducted using an apparatus Q2500 DSC (TA Instruments, New Castle, DE, USA) for the prepolymer SK1_Sn with varying amounts of the photoinitiator Omnirad 2022 (0.5 wt%, 1 wt%, 1.5 wt%, 2 wt%, 2.5 wt%). Measurements were performed at three different light intensities (20, 100, and 500 mW/cm^2^) in nitrogen atmosphere at 25 °C. Each measurement was conducted in two cycles. The measurement time was 10 min for each composition. The obtained results are summarized in Figure 5, Figure 6, Figure 7 and Figure 8 for the light intensities 20, 100 and 500 mW/cm^2^, respectively, and all thermograms of the measured samples are presented in Supplementary Information (see in SI Figures S1–S15).

**Figure 9:**
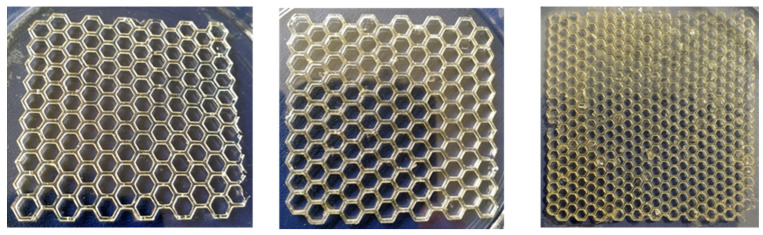
Examples of meshes formed from PhotoBioCure^®^ material using molds prepared from matrices made with SLA technology.

The measurements were conducted across the entire wavelength spectrum (without using filters). For all compositions, the peak maximum was observed after approximately 18 s. The calculated degree of conversion indicated that a concentration of 1 wt% of the photoinitiator was the most effective at reaching 100% conversion, irrespective of the irradiation intensity. It can be seen in Figure 9 that conversion percentage was decreased with increasing concentration of a photoinitiator. The phenomenon can be ascribed to several factors. Excessive amounts of photoinitiators cause the inner filter effect, where excessive light absorption near the surface reduces penetration into deeper layers, limiting polymerization [53,54]. Additionally, higher concentrations generate more reactive species, increasing bimolecular termination reactions, which reduce the number of radicals available for propagation. This imbalance, along with potential side reactions and system saturation, disrupts the polymerization process, leading to lower monomer-to-polymer conversion efficiency [55,56,57]. An analysis of the effect of light intensity determines that the lower conversion efficiency was observed at higher light intensities. It can be due to the overgeneration of reactive species, leading to increased termination reactions. Additionally, excessive light intensity can cause thermal effects, photoinitiator degradation, and oxygen inhibition, all of which disrupt the polymerization process [58].

### 3.7. Gel Fraction of Thin Films

The gel fractions data are collected in Table 4. The gel fraction results for the tested macromonomers, including SK1_Sn, SK2_Sn, and SK3_Sn, range between 87.9% and 88.5%, with the commercial PhotoBioCure^®^ exhibiting a similar gel fraction of 88.4%. These consistent values across all samples suggest a high degree of crosslinking, indicating that the photocuring process was effective in forming a stable network structure. There is a small difference in the gel fraction between the macromonomers and the reference material, despite the differences in dynamic viscosity measured at 25 °C. In general, the higher the content of the gel fraction, the greater the restrictions on the movement of the polymer chains, leading to higher dynamic viscosity, as observed for the SK series. However, the results for the reference material do not correlate with this relationship, which may be related to the details of the unknown chemical structure.

### 3.8. Mechanical Analysis

The mechanical properties of the obtained thin samples were tested on an Instron 3366 testing machine. For each material, at least 10 samples were tested. The samples with extreme properties were excluded from the statistics and average values with standard deviations are presented in Table 5. During the test, the following properties were directly measured: strain at break, tensile strength and elastic modulus at strains between 5% and 6%. These properties are important for the mechanical behavior of the materials after the implantation in the body [59].

Mechanical analysis showed that the tensile strength, modulus and elongations at break for SK_Sn materials are similar to PhotoBioCure^®^ material. The slight differences that occurred in mechanical properties may be caused by small variations in gel fractions. Overall, elongation was measured up to 110%, tensile strength was up to 1.9 MPa and moduli at a 2–3% strain and a 5–6% strain were in the range of 3–4 MPa. The tested materials all exhibit elongations at breaking point that are comparable to or above 100%, which is a desirable value for the medical applications, especially for soft tissue of the abdominal wall [60].

According to the literature [26,40,41], an elongation of around 40% is sufficient for meshes. The tensile strength values for various materials are available on the market, with tensile strengths between 0.760 and 13.7 MPa [39,61,62]. These values can be directly compared with those obtained in our tests, as they were determined using the same tensile test setup. The lists of modulus values for abdominal wall tissues were at levels of 40–80 kPa [40]. This is a very advantageous combination of mechanical properties, because an increase in modulus typically reduces a material’s flexibility. This means that the material can withstand high loads (compared to other tested materials) while also having a large capacity for deformation without risking rupture.

### 3.9. Mesh Manufacturing

The various designs for positive matrices were prepared, incorporating wall tapering and various mesh sizes (up to 4 mm). Several experimental positive matrices were produced from UV-curable reactive resin using the SLA (LCD variant) technique. Compared to FDM 3D printing, the applied technology for creating positive matrices allows for prints (matrices) with better detail and higher surface density. Experimental production molds were made from these matrices, and trial mesh castings were created from the material that showed the best mechanical properties within SK series and with PhotoBioCure^®^. The mesh manufacturing was carried out at 23 °C. The obtained results, mostly correctly formed meshes, allow the conclusion that the adopted technological and tooling concept is suitable for forming this type of product. The examples of meshes formed using molds prepared from matrices made with SLA technology are shown in Figure 9. The developed procedure allows the final product to be easily and quickly cast from a reactive macromer in an open, flexible rubber mold using the UV curing technique, followed by product demolding. The production mold is reusable multiple times, having been checked after each molding cycle.

### 3.10. Cell Viability

**Figure 10:**
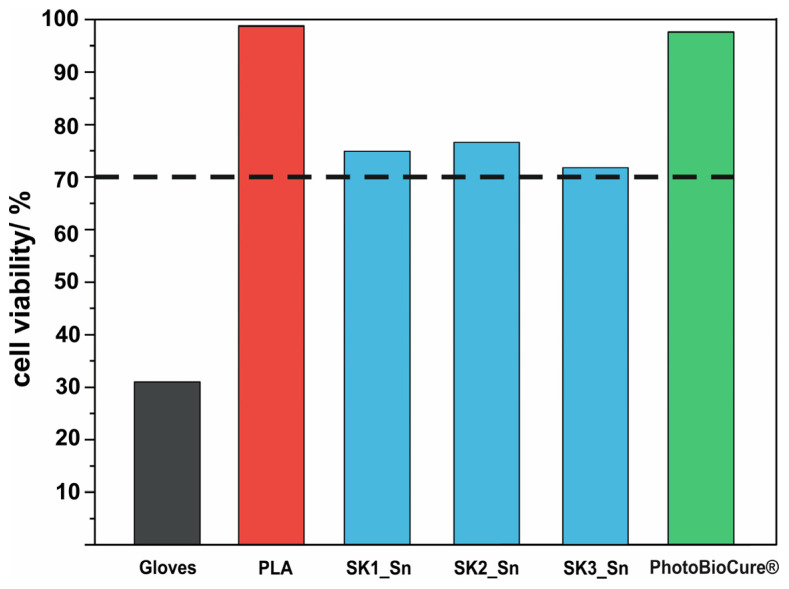
L929 cell viability after 24 h of exposure to extracts of SK and PhotoBioCure^®^ materials. The dashed line indicates the 70% cytotoxicity threshold according to ISO 10993-5.

We assessed materials cytotoxicity in accordance with ISO 10993-5 to determine their suitability for biomedical applications. The cell viability results for L929 mouse fibroblasts exposed to material extracts for 24 h are shown in Figure 10. As expected, all materials of SK series resulted in high cell viability without further postprocessing. DBTDL, a known organotin catalyst, is notoriously difficult to remove from polymers during purification [27]. According to the literature, DBTDL has an IC50 of approximately 3 µg/mL [63], making it more toxic than other weak Lewis acids or metal salts. We estimate the residual catalyst content in our test samples to be between 100 and 150 µg, but all tested materials show levels of cell viability that are above the threshold (70% cell viability according ISO 10993-5), with the highest values being for PhotoBioCure^®^. The observed differences in cell viability can arise from differences in the chemistries of tested materials and the reference.

## 4. Conclusions

In this study, difunctional methacrylate monomers were successfully synthesized, optimized and characterized for photopolymerization. The synthesis process was monitored by infrared spectroscopy and evaluated through nuclear magnetic resonance and gel permeation chromatography, and confirmed the desired molecular structure and molecular weight of synthesized monomers (Mn ~7000 g/mol). Photo-DSC revealed that the photoinitiator concentration and light intensity had a strong effect on monomer conversion. The highest, 100%, conversion was reached for 1 wt% of photoinitiator, irrespective of the irradiation dose. Mechanical testing of the photocured elastomeric films demonstrated properties aligned with the characteristics of soft tissue, including tensile strength which was measured up to 3.5 MPa and modulus between 3 and 4 MPa. Furthermore, the 3D printability was verified, underscoring the versatility of this approach in creating tailored biomedical constructs. Although in vitro cytotoxicity testing revealed superior cell viability for the PhotoBioCure^®^ reference material (97%) compared to the tin-catalyzed systems (73%), this research underscores the promise of difunctional methacrylate monomers as a flexible, biocompatible solution for biomedical applications. Future work will focus on assessing the long-term stability, biodegradability and in vivo performance of these materials. Enhancing their functionality through the incorporation of bioactive molecules or nanoparticles could expand their applications in chronic wound healing, inguinal hernia and orthopedic repair. Additionally, further studies with diverse cell types and biological environments will be essential to confirm their clinical potential.

## Figures and Tables

**Table 1:** Summary of the reaction parameters.

Materials	Catalyst Concentration (I Step) [mmol]	I Step [hours]	Catalyst Concentration (II Step) [mmol]	II Step [hours]	Total [hours]	Reaction Yield [%]
	DABCO		DBTDL			
SK1_Sn	0.21	71	0.1	24	95	65
SK2_Sn	0.21	71	0.1	24	95	63
SK3_Sn	0.21	73	0.1	25	98	68

**Table 2:** GPC analysis results of the obtained macromonomers.

Sample	M_n_ (g/mol)	M_w_ (g/mol)	PDI
SK1_Sn	7000	11,600	1.67
SK2_Sn	7300	12,000	1.64
SK3_Sn	7400	12,900	1.74
PhotoBioCure^®^	7000	11,500	1.65

**Table 3:** Dynamic viscosity of the obtained macromonomers.

Synthesis	Dynamic Viscosity [mPa·s] ƴ = 0.1 [rpm], T = 25 °C
SK1_Sn	249.000
SK2_Sn	238.100
SK3_Sn	233.800
PhotoBioCure^®^	161.700

**Table 4:** Gel fraction of the produced thin films.

Samples	Gel Fraction (%)
SK1_Sn	88.1
SK2_Sn	88.5
SK3_Sn	87.9
PhotoBioCure^®^	88.4

**Table 5:** Summary of the average mechanical properties of materials.

Material	σ_br_ (kPa)	ε_br_ (%)	E @ 2–3% (kPa)	E @ 5–6% (kPa)	Stiffness (N/cm)	Strength (Max Stress) (N/cm)
SK1_Sn	1640 ± 221	110 ± 10	4080 ± 855	3660 ± 763	21.2 ± 5.4	9.4 ± 1.4
SK2_Sn	1760 ± 557	109 ± 15	3720 ± 948	3890 ± 754	21.2 ± 6.6	10.3 ± 2.3
SK3_Sn	1410 ± 262	104 ± 18	3840 ± 808	3710 ± 952	21.4 ± 6.2	8.1 ± 1.8
PhotoBioCure	1910 ± 384	105 ± 13	3780 ± 729	3640 ± 497	21.2 ± 2.8	11.1 ± 1.8

## Data Availability

The data presented in this study are available on request from the corresponding author. The data are not publicly available due to privacy [insert reason here].

## Supplementary Materials

The following supporting information can be downloaded at: www.mdpi.com/article/10.3390/polym16243584/s1, Figure S1: The thermogram of the FotoDSC for SK1_Sn with 0.5% Omnirad 2022 crosslinked at an intensity of 20 mW/cm^2^, Figure S2: The thermogram of the FotoDSC for SK1_Sn with 1% Omnirad 2022 crosslinked at an intensity of 20 mW/cm^2^, Figure S3: The thermogram of the FotoDSC for SK1_Sn with 1.5% Omnirad 2022 crosslinked at an intensity of 20 mW/cm^2^, Figure S4: The thermogram of the FotoDSC for SK1_Sn with 2% Omnirad 2022 crosslinked at an intensity of 20 mW/cm^2^, Figure S5: The thermogram of the FotoDSC for SK1_Sn with 2.5% Omnirad 2022 crosslinked at an intensity of 20 mW/cm^2^, Figure S6: The thermogram of the FotoDSC for SK1_Sn with 0.5% Omnirad 2022 crosslinked at an intensity of 100 mW/cm^2^, Figure S7: The thermogram of the FotoDSC for SK1_Sn with 1% Omnirad 2022 crosslinked at an intensity of 100 mW/cm^2^, Figure S8: The thermogram of the FotoDSC for SK1_Sn with 1.5% Omnirad 2022 crosslinked at an intensity of 100 mW/cm^2^, Figure S9: The thermogram of the FotoDSC for SK1_Sn with 2% Omnirad 2022 crosslinked at an intensity of 100 mW/cm^2^, Figure S10: The thermogram of the FotoDSC for SK1_Sn with 2.5% Omnirad 2022 crosslinked at an intensity of 100 mW/cm^2^, Figure S11: The thermogram of the FotoDSC for SK1_Sn with 0.5% Omnirad 2022 crosslinked at an intensity of 500 mW/cm^2^, Figure S12: The thermogram of the FotoDSC for SK1_Sn with 1% Omnirad 2022 crosslinked at an intensity of 500 mW/cm^2^, Figure S13: The thermogram of the FotoDSC for SK1_Sn with 1.5% Omnirad 2022 crosslinked at an intensity of 500 mW/cm^2^, Figure S14: The thermogram of the FotoDSC for SK1_Sn with 2% Omnirad 2022 crosslinked at an intensity of 500 mW/cm^2^, Figure S15: The thermogram of the FotoDSC for SK1_Sn with 2.5% Omnirad 2022 crosslinked at an intensity of 500 mW/cm^2^.
