# Supplementary material for: Synthesis and Characterization of Photocurable Difunctional Monomers for Medical Applications
Source: Polymers (Basel). 2024 Dec 21;16(24):3584. doi: 10.3390/polym16243584 (PMC11678372; doi:10.3390/polym16243584)
Supplement: Supplementary file 1 [file polymers-16-03584-s001.zip › polymers-3347558-supplementary.pdf]

Synthesis and Characterization of Photocurable Difunctional Monomers for Medical Applications

Gokhan Demirci[1,2], Agata Goszczyńska[1,2], Martyna Sokołowska[1,2], Marek Żwir[1], Krzysztof Gorący[1], Miroslawa El Fray[1]*

[1]Department of Polymer and Biomaterials Science, Faculty of Chemical Technology and Engineering, West Pomeranian University of Technology in Szczecin,
Al. Piastów 45, 70-311 Szczecin, POLAND

[2]Poltiss Spółka Z Ograniczoną Odpowiedzialnością, Al. Wojska Polskiego 150/171-324 Szczecin, Poland

Supplementary Materials

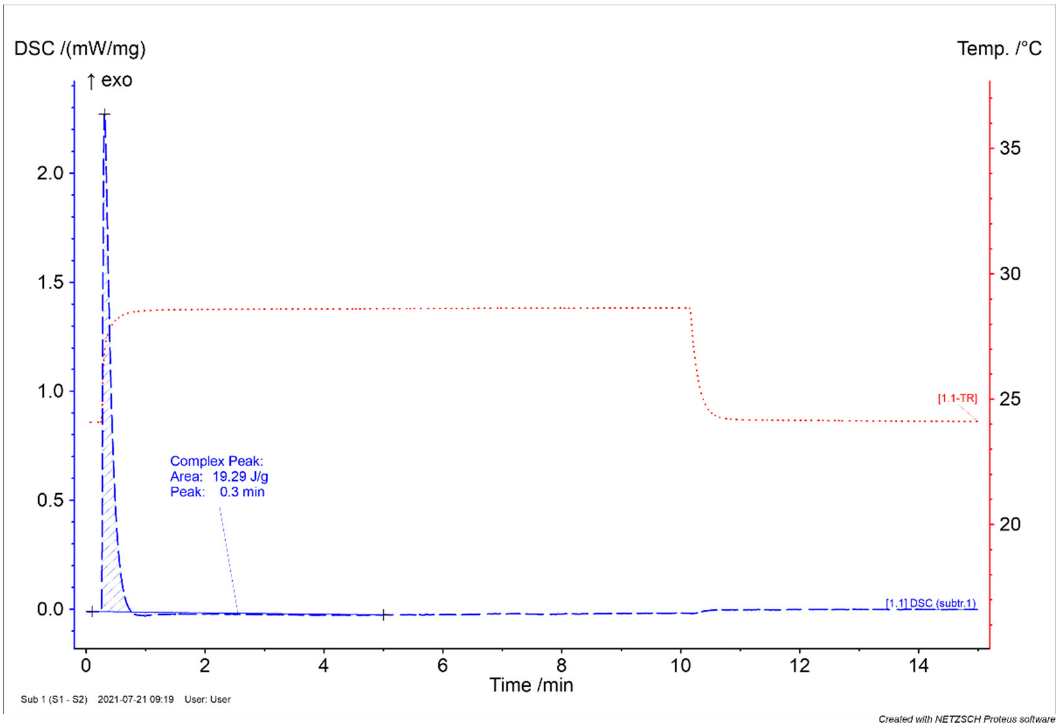

***Figure S1** The thermogram of the FotoDSC for SK1_Sn with 0.5% Omnirad 2022 crosslinked at an intensity of 20 mW/cm²*

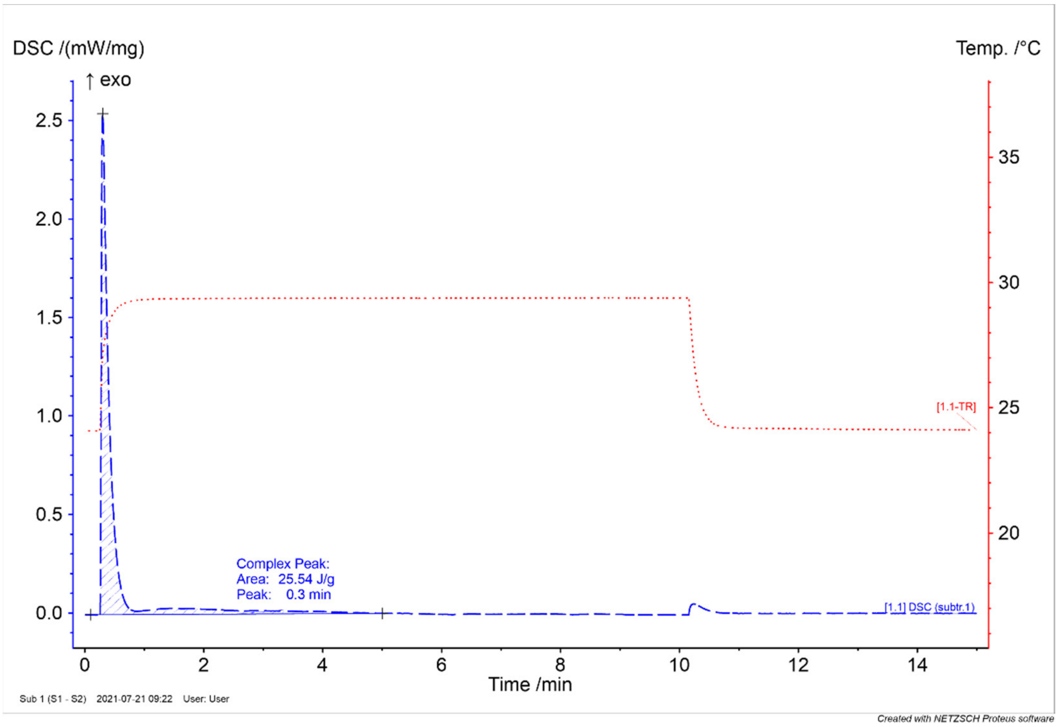

Figure S2 *The thermogram of the FotoDSC for SK1_Sn with 1% Omnirad 2022 crosslinked at an intensity of 20 mW/cm²*

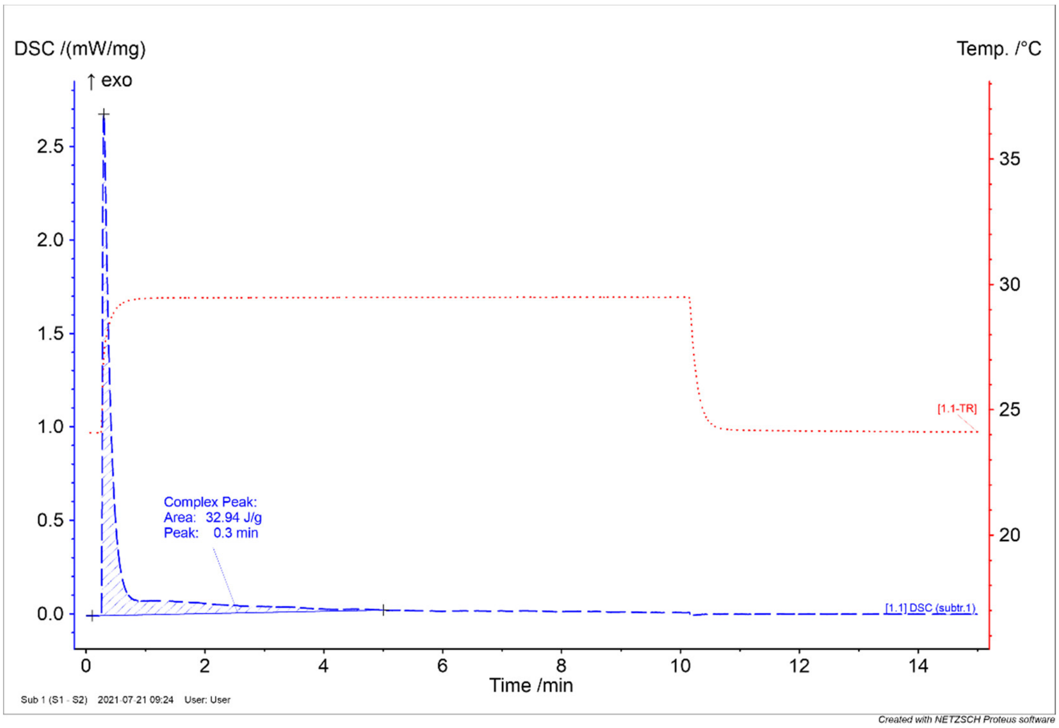

Figure S3 *The thermogram of the FotoDSC for SK1_Sn with 1.5% Omnirad 2022 crosslinked at an intensity of 20 mW/cm²*

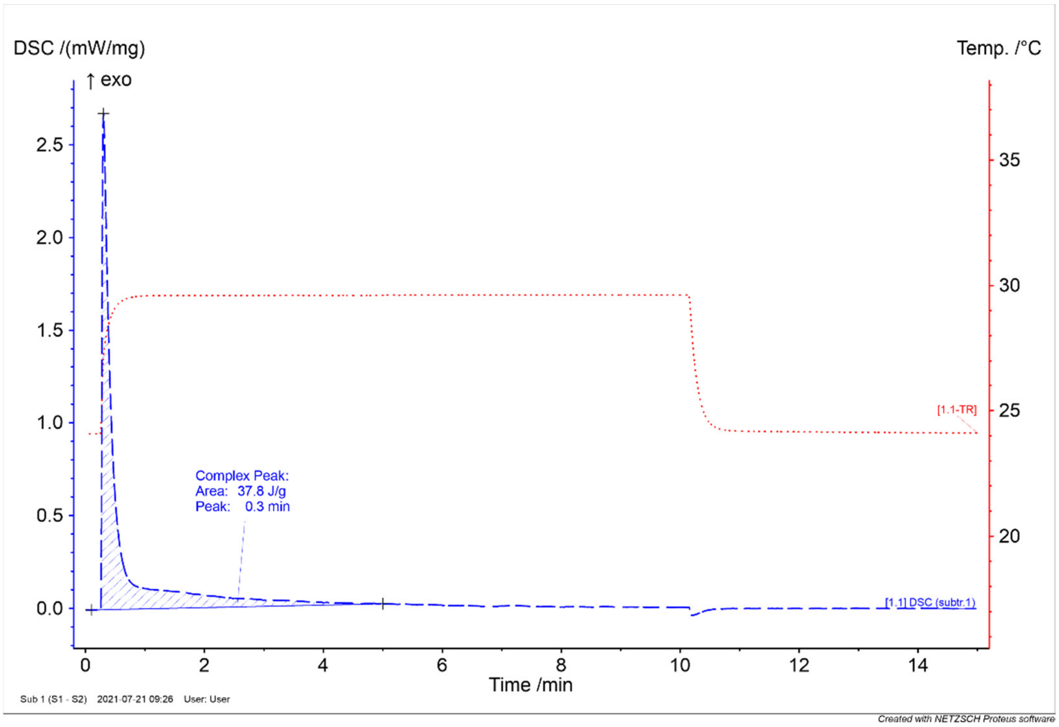

Figure S4 *The thermogram of the FotoDSC for SK1_Sn with 2% Omnirad 2022 crosslinked at an intensity of 20 mW/cm²*

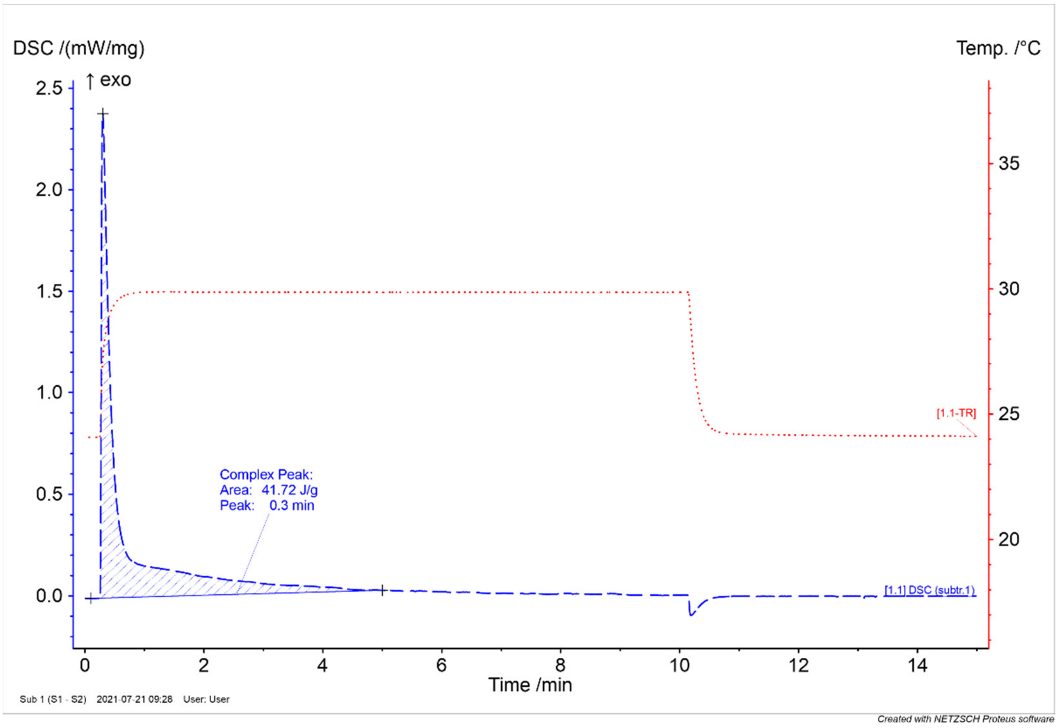

Figure S5 *The thermogram of the FotoDSC for SK1_Sn with 2.5% Omnirad 2022 crosslinked at an intensity of 20 mW/cm²*

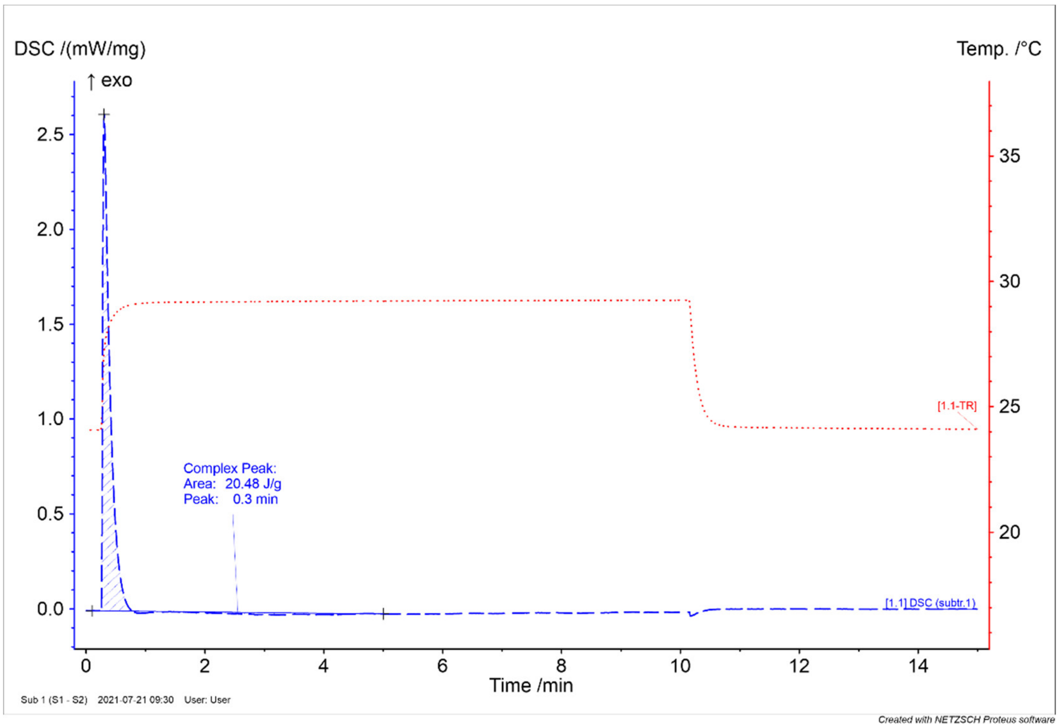

Figure S6 *The thermogram of the FotoDSC for SK1_Sn with 0.5% Omnirad 2022 crosslinked at an intensity of 100 mW/cm²*

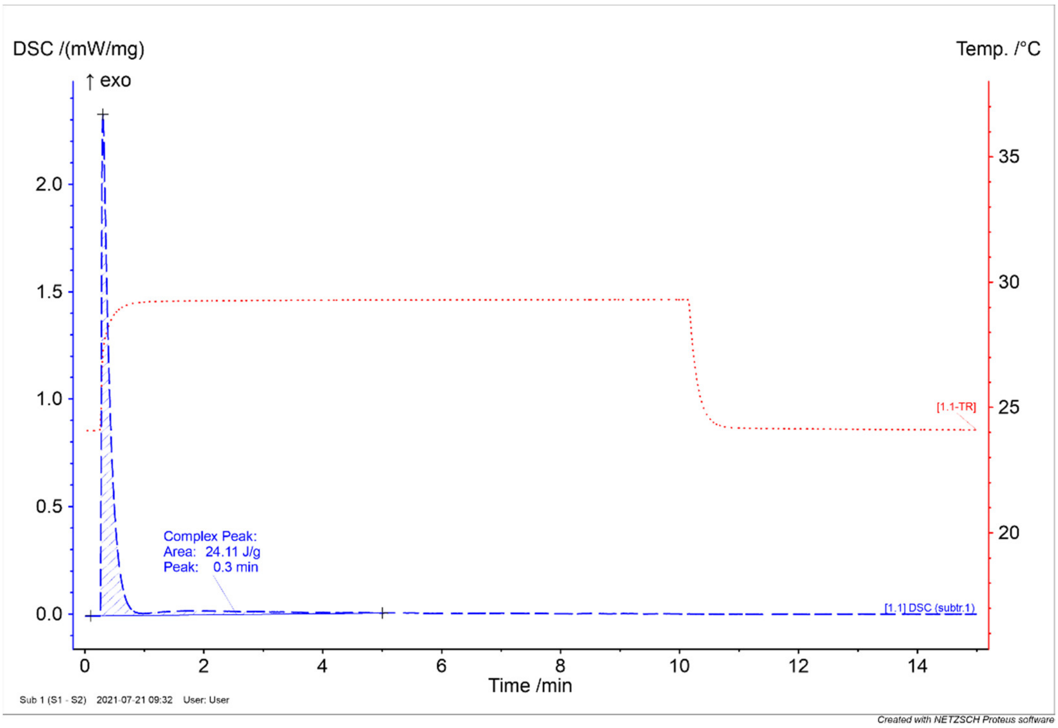

Figure S7 *The thermogram of the FotoDSC for SK1_Sn with 1% Omnirad 2022 crosslinked at an intensity of 100 mW/cm²*

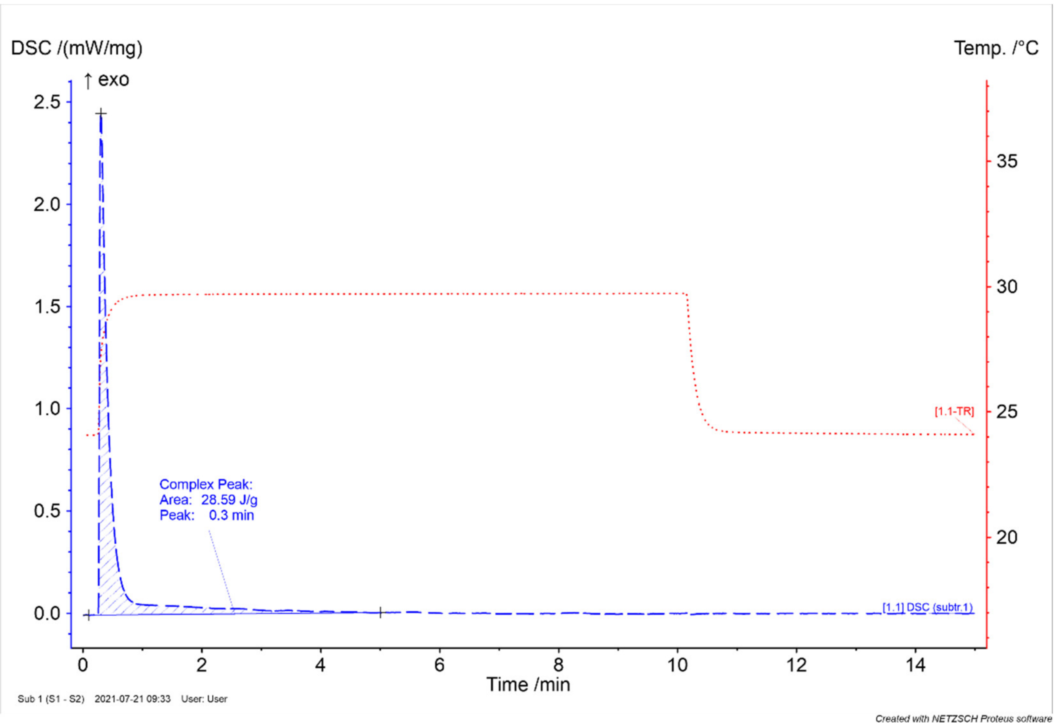

Figure S8 *The thermogram of the FotoDSC for SK1_Sn with 1.5% Omnirad 2022 crosslinked at an intensity of 100 mW/cm*^{2}

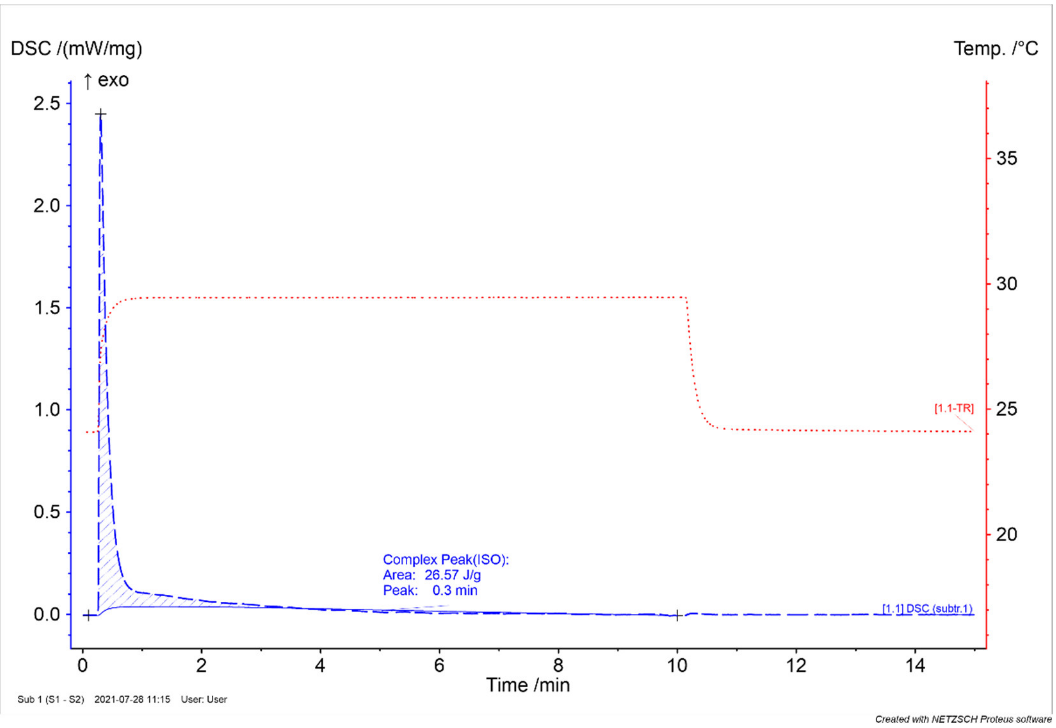

Figure S9 *The thermogram of the FotoDSC for SK1_Sn with 2% Omnirad 2022 crosslinked at an intensity of 100 mW/cm*^{2}

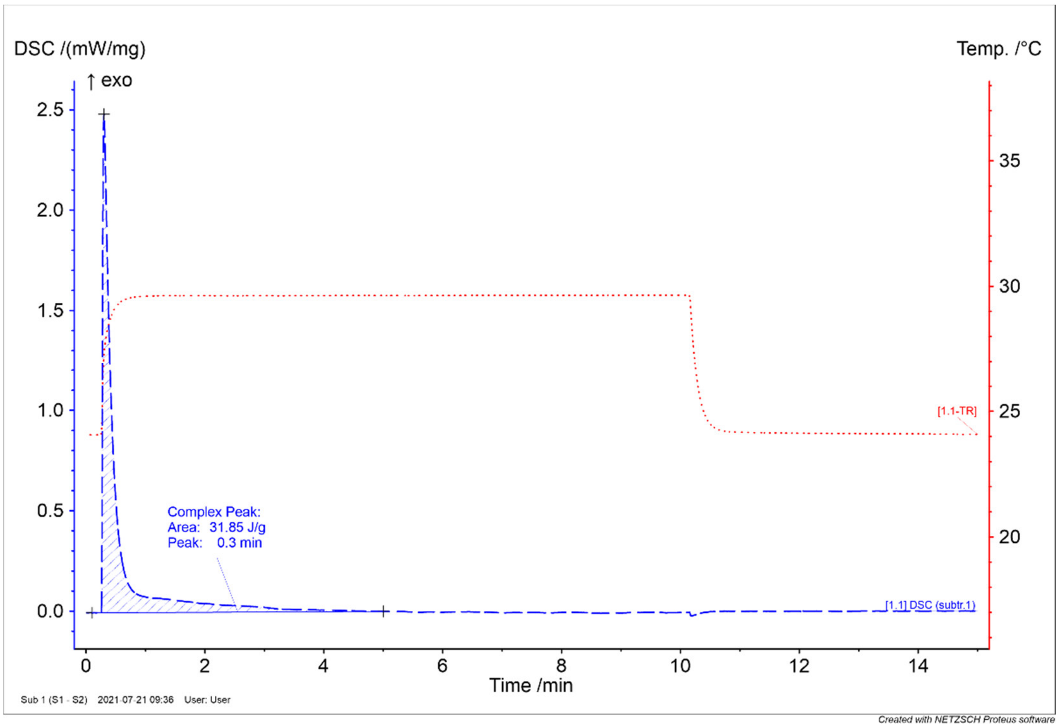

Figure S10 *The thermogram of the FotoDSC for SK1_Sn with 2.5% Omnirad 2022 crosslinked at an intensity of 100 mW/cm²*

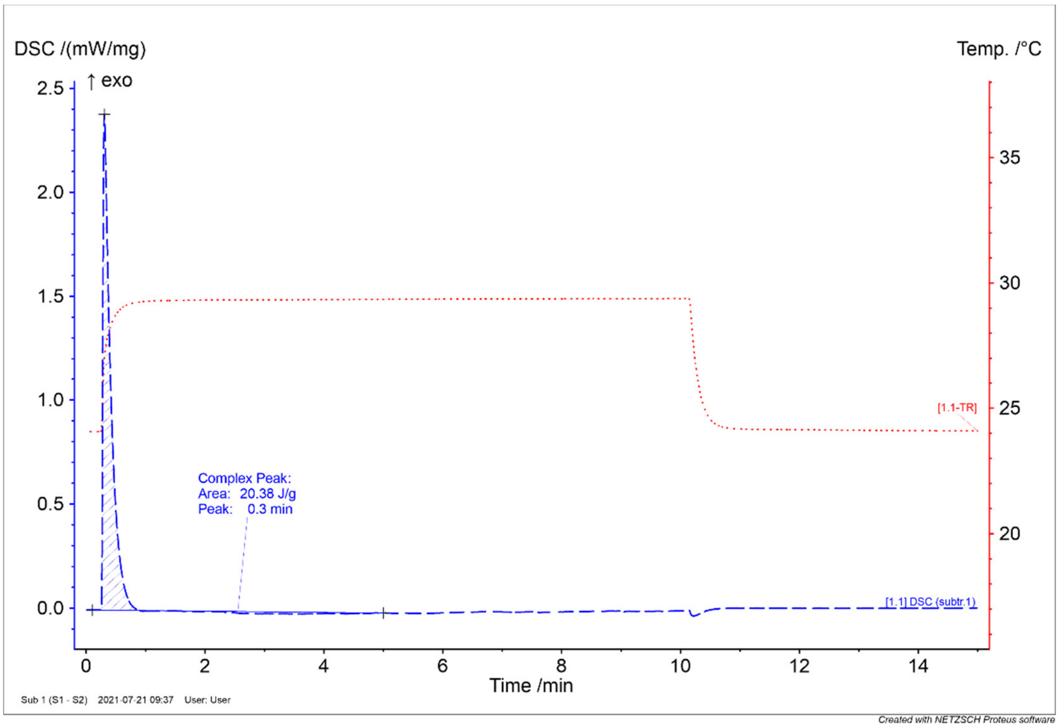

Figure S11 *The thermogram of the FotoDSC for SK1_Sn with 0.5% Omnirad 2022 crosslinked at an intensity of 500 mW/cm²*

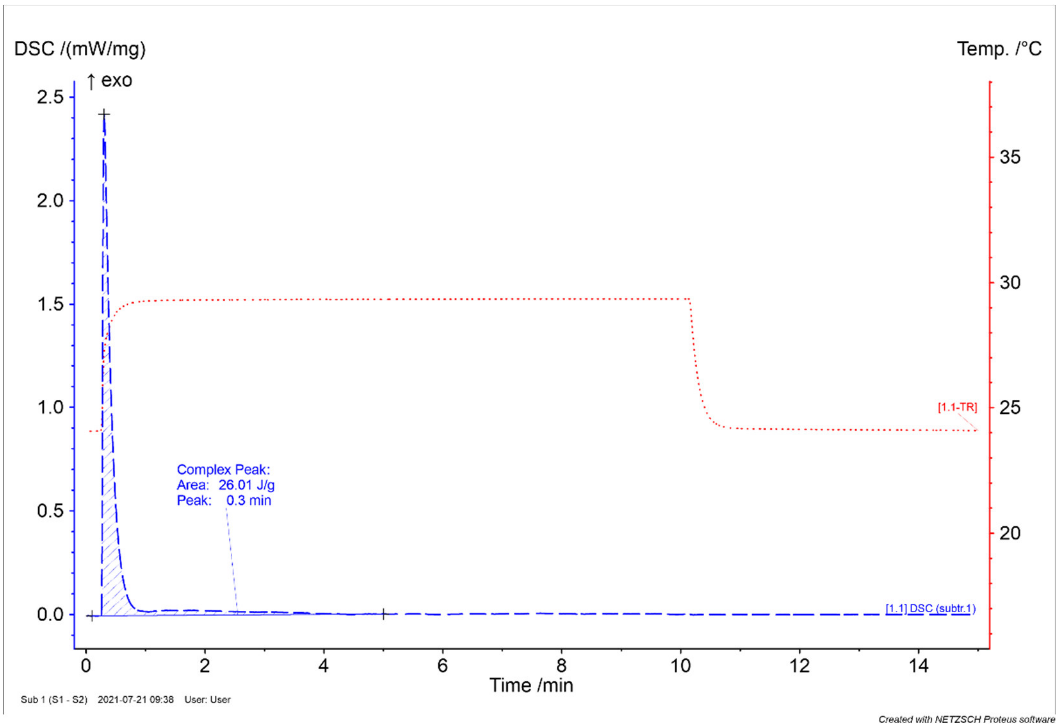

Figure S12 *The thermogram of the FotoDSC for SK1_Sn with 1% Omnirad 2022 crosslinked at an intensity of 500 mW/cm²*

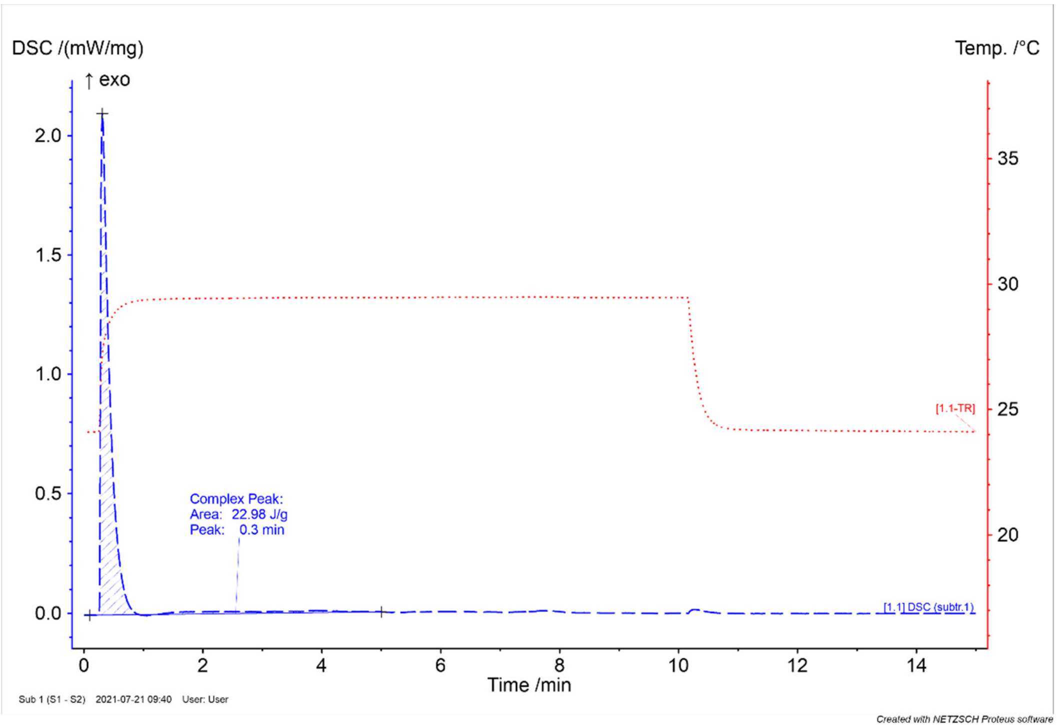

Figure S13 *The thermogram of the FotoDSC for SK1_Sn with 1.5% Omnirad 2022 crosslinked at an intensity of 500 mW/cm²*

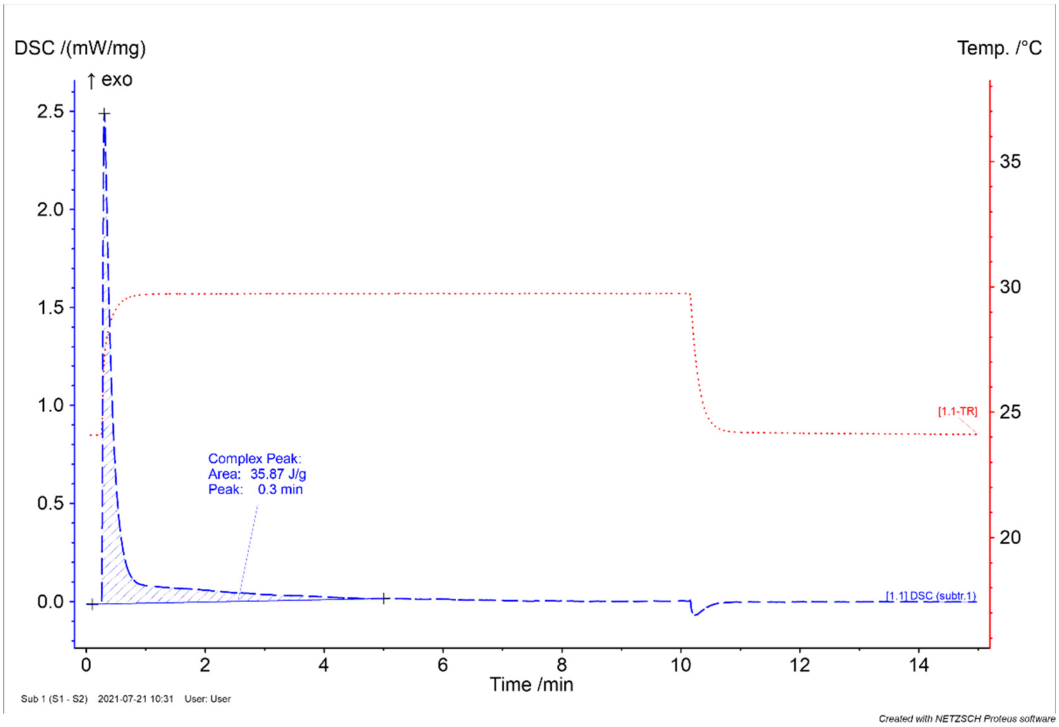

Figure S14 *The thermogram of the FotoDSC for SK1_Sn with 2% Omnirad 2022 crosslinked at an intensity of 500 mW/cm²*

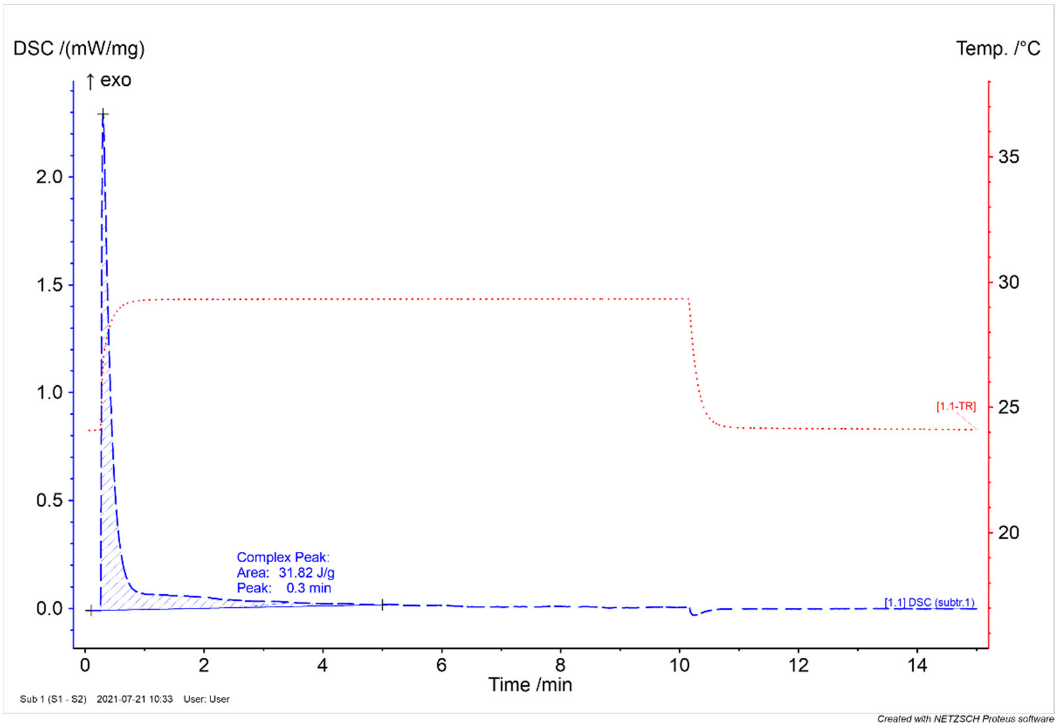

Figure S15 *The thermogram of the FotoDSC for SK1_Sn with 2.5% Omnirad 2022 crosslinked at an intensity of 500 mW/cm²*
